# Meta-Analysis of Positive Psychology Interventions on the Treatment of Depression

**DOI:** 10.7759/cureus.21933

**Published:** 2022-02-05

**Authors:** Shannon Pan, Kiran Ali, Chanaka Kahathuduwa, Regina Baronia, Yasin Ibrahim

**Affiliations:** 1 Psychiatry, Texas Tech University Health Sciences Center, Lubbock, USA

**Keywords:** well-being, optimism, gratitude, psychotherapy, treatment of depression, depression, positive psychology

## Abstract

This meta-analysis examined the efficacy of positive psychology interventions (PPIs) in treating depression in 11 articles. PubMed, Web of Science, and Clinical Key were used to identify papers published from 2010 to 2020 that utilized PPIs. Key terms were “positive psychology” and “treatment of depression.” Studies on adults with (a) depressive symptoms or (b) diagnosed clinical depression were included. A random-effects model was used to compare PPIs and control groups on post- vs. pre-intervention differences in depression scores. Data analysis examined Beck Depression Inventory-II (BDI-II), Center for Epidemiologic Studies Depression Scale (CES-D), and Quick Inventory of Depressive Symptomatology-Self-Report (QIDS-SR16) scores. Findings show PPIs are effective in treating depressive symptoms, with significant improvements in depression scores when compared to control groups in all but one study. This was true for both post- vs. pre-intervention (pooled Cohen’s d = −0.44 (−0.77, −0.11)) and follow-up- vs. pre-intervention analyses (pooled Cohen’s d = −0.46 (−1.02, 0.09)). PPIs can improve the accessibility and affordability of depression treatments.

## Introduction and background

Depression is a global crisis. The World Health Organization estimates that a staggering 264+ million people suffer from depression, and with this comes the enormous social, economic, and systemic burdens that societies must face [1]. While medication can be effective in lessening acute symptoms, more long-term solutions require extensive psychotherapy so individuals can learn healthy and sustainable coping mechanisms [2].

There is a range of psychotherapy techniques that are used to treat depression, such as cognitive-behavioral therapy (CBT), interpersonal therapy (IPT), and third-wave cognitive and behavior therapies such as dialectical behavioral therapy (DBT) and acceptance and commitment therapy (ACT) [3,4]. More recently, positive psychotherapy (PPT) has gained increased attention as a possible treatment modality.

Positive psychology terminology

**Figure 1:**
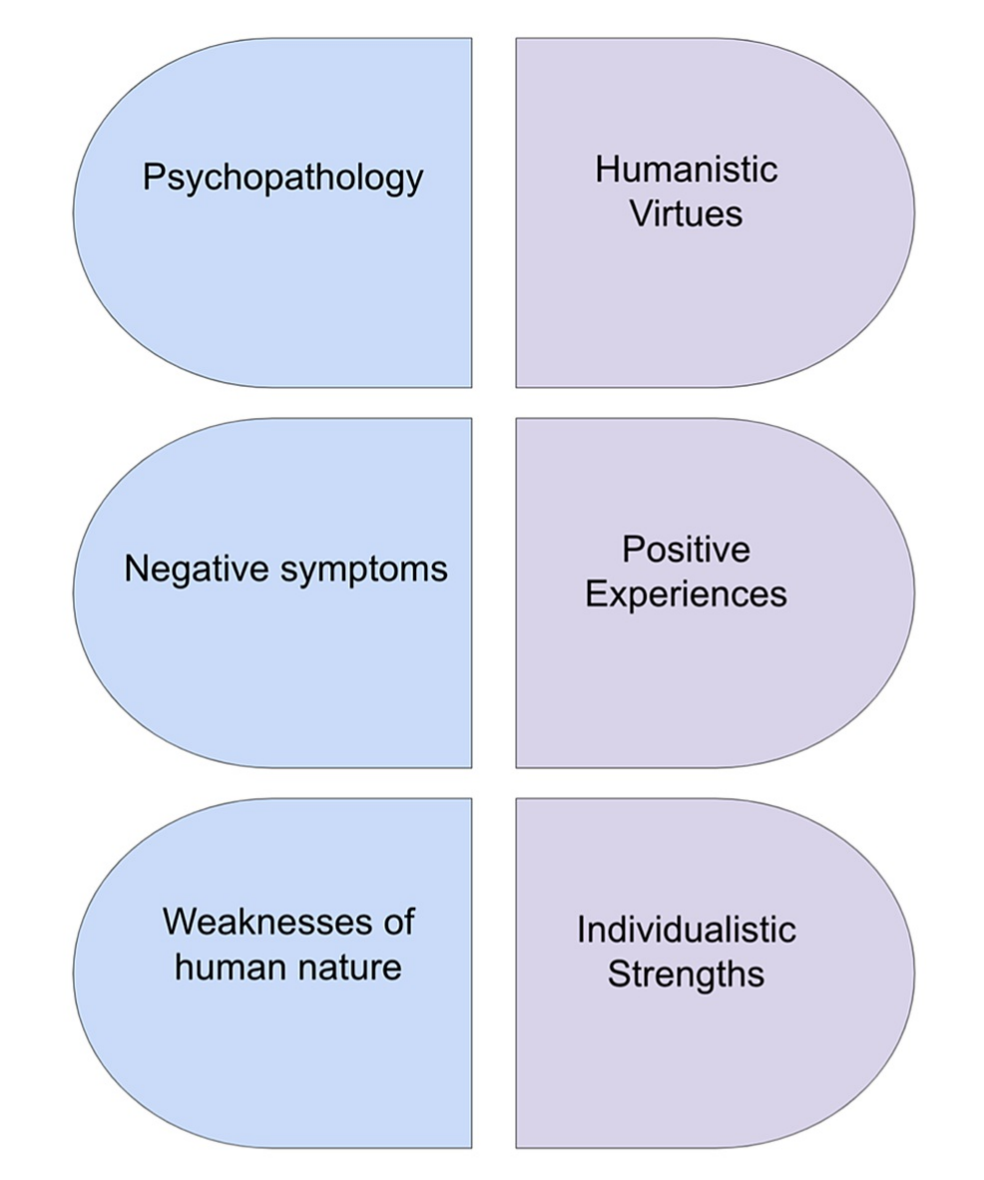
Balance of views. Positive psychology provides a balance to the previous views of psychology; it gives reasons that make life worth living and fulfilling. Life consists of both suffering and joy, and positive psychology serves as a reminder that the focus should not be one-sided.

Positive psychology is an umbrella term that encompasses a wide range of humanistic theories and principles [5]. There is no singular definition, but its origins are from Martin Seligman, President of the American Psychological Association in 1998 and pillar of the positive psychology movement [5]. Prior to this movement, the main focus of mental health treatments was on the pathology of negative symptoms, and most of the efforts were channeled toward reducing these symptoms [6]. However, Seligman encouraged a more holistic approach to treating psychopathology - he emphasized the importance of focusing on positive aspects of life, such as love, gratitude, social ties, humor, and resilience (Figure 1) [7,8].

**Figure 2:**
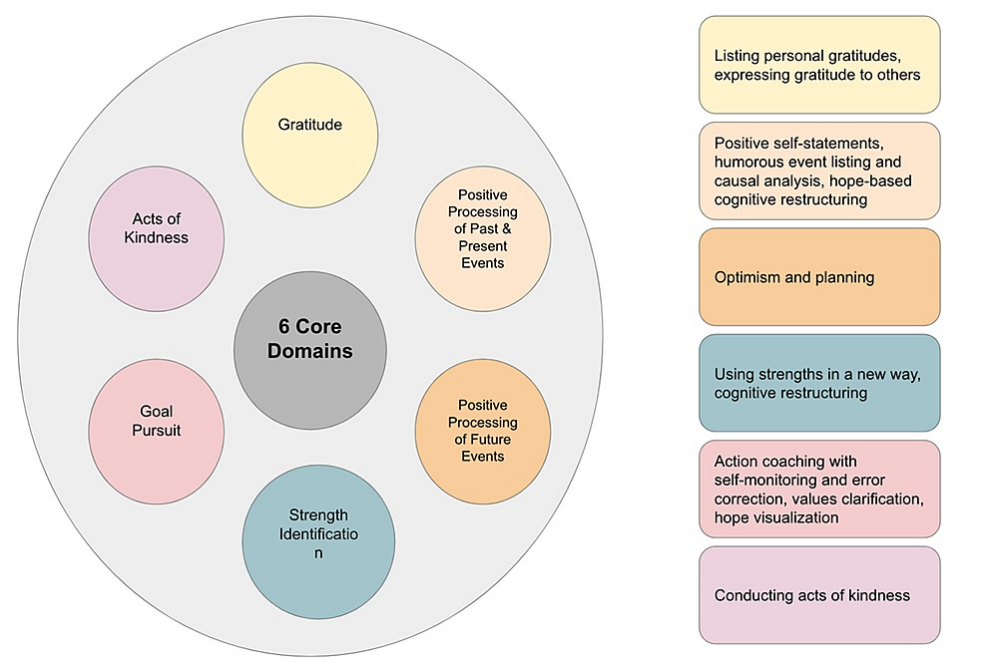
Six core domains of positive psychology. A color-coded diagram of the six core domains of positive psychology. Adapted from the topographical map by Gorlin et al. [9]. The subdomains on the left represent more specific examples and activities that are commonly used in positive psychology interventions.

**Figure 3:**
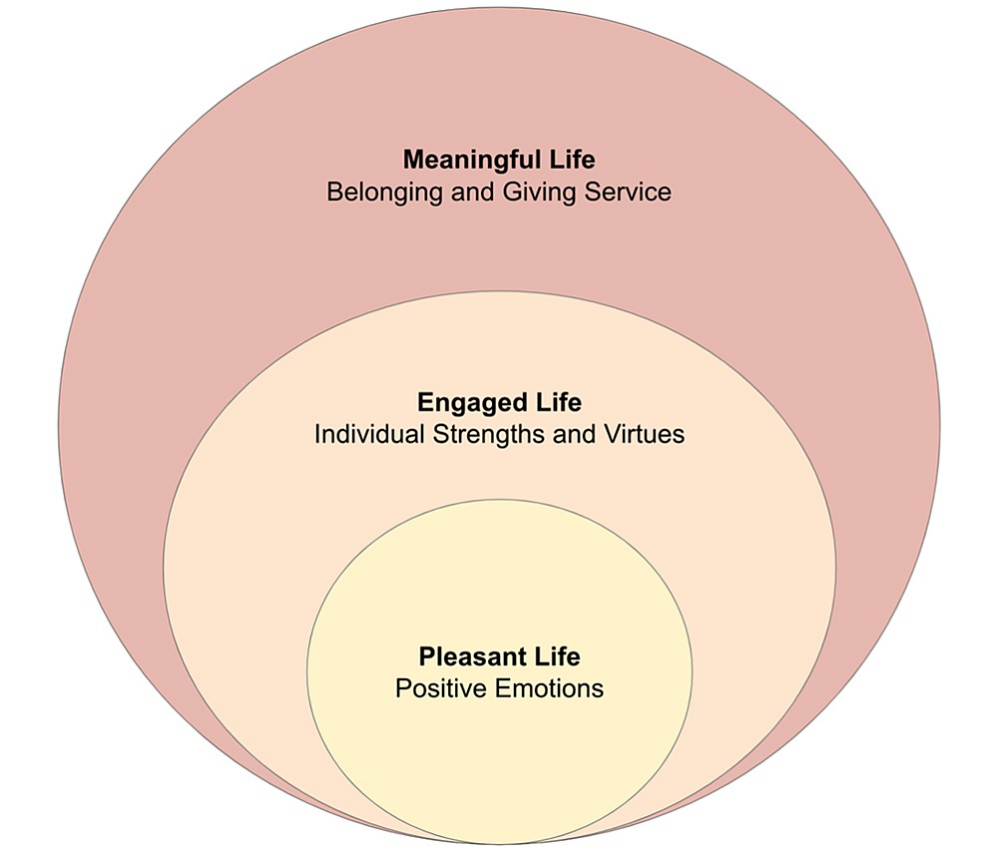
Theory of authentic happiness. A diagram based on the three components of a good life as described in Schrank et al. [5]. This illustrates how Seligman’s theory on authentic happiness spans across many domains in an individual’s life, ranging from a more personal to a societal level. As depicted, happiness tends to radiate outwards when it starts from within.

Positive psychology interventions (PPIs) can be categorized into six core domains: acts of kindness, gratitude, positive processing of past and present events, positive processing of future events, goal pursuit, and strength identification [9]. Each core domain is represented by an intervention (see Figure 2). While there are many more subdomains, the overall goal of these domains is to help individuals find meaning and purpose in their lives based on their own unique strengths [10]. This aligns with Seligman’s theory on what constitutes authentic happiness, which includes positive emotions, engagement, and meaning (as seen in Figure 3) [5].

PPIs aim to improve well-being at not only the individual level but at the societal and interpersonal levels as well [5]. Although research showing empirical evidence of the efficacy of PPI is still recent, there have already been many studies that show PPIs are effective in combating symptoms of depression, such as suicidality and hopelessness [11-13].

PPT is the psychotherapy technique that emerged from the movement and represents a combination of evidence-based interventions combined into a single intervention manual [5]. PPT allows for a more uniform way of delivering PPT exercises to patients, and since it was originally developed for people with depressive symptoms, it serves as a promising intervention for more serious disorders such as depression [5]. A distinguishing feature of PPT, compared with other psychotherapy techniques, such as CBT, is its emphasis on fostering traits unique to each individual that allow them to live an optimal life [14-16]. Patients are encouraged to practice mindfulness and awareness, and in doing so, they can increase self-awareness, positive emotions, and ultimately well-being [17,18].

Purpose of study

The purpose of this study was to systematically analyze the effects of PPI on the treatment of depression in adults and the elderly. Studies within the last 10 years that utilize PPIs on patients with depression or depressive symptoms were examined. This meta-analysis will provide researchers with empirical evidence of the intervention’s benefits, specifically its therapeutic value, cost-effectiveness, and longitudinal benefits. We hypothesized that PPIs would effectively treat depression or alleviate depressive symptoms when compared to control groups.

## Review

Methods

Search Strategy and Eligibility Screening

Three databases, PubMed, Web of Science, and Clinical Key, were used to identify papers published from 2010 to 2020, and hand searches were also conducted after the systematic searches. The key terms used for each database were “positive psychology” and “treatment of depression.”

**Figure 4:**
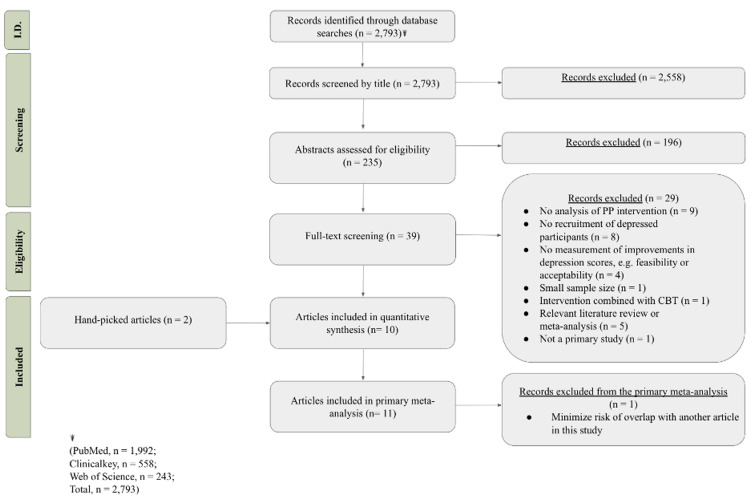
Flow diagram. I.D.: identification phase; PP: positive psychology; CBT: cognitive-behavioral therapy.

Only studies with a patient population of adults aged 18+ years with either (a) depressive symptoms or (b) diagnosed clinical depression were included in the analysis. Studies were included if a PPI was used. There were restrictions on study design and outcome measurement. This paper does not include positive intervention in conjunction with already-proven psychotherapies such as CBT and meditation. Studies that included psychopharmacological intervention were included if both of intervention group and control group received the same medication. Opinion pieces such as editorials, abstracts, and letters to the editor were not included. These exclusion criteria were applied during the title and abstract screening process. Papers were all published in English (Figure 4).

Data Extraction

Data were extracted from the eligible manuscripts into pre-defined data fields. Sample size, the scale used to measure depression symptoms/severity, mean post- vs. pre-intervention change of depression scores, and standard deviation of post- vs. pre-intervention change of depression scores of interventions (i.e., positive psychology) and control groups were extracted from the eligible full-text articles. When only the standard deviations of marginal means were available, the standard deviations of mean differences were imputed using the methods described in the Cochrane Handbook [19]. In addition, the following data of the intervention group were extracted as potential covariates of the above outcomes: mean age of participants, the proportion of females, the proportion of participants who received >12 years of education, the proportion of participants who were employed, and the proportion of participants who had a partner/were married. Furthermore, the year of publication and duration of intervention were extracted as potential covariates. When the articles reported outcomes of delayed follow-up following an intervention, the follow-up vs. pre-intervention change of depression scores and standard deviation of post- vs. pre-intervention change of depression scores of interventions (i.e., positive psychology) and control groups were extracted. The duration of follow-up was also extracted from these articles as an additional covariate.

Data Analysis

Two separate DerSimonian-Laird random-effects meta-analyses were performed using the "meta" package (version 4.11-0) in R statistical software (version 3.6.2; R Foundation for Statistical Computing, Vienna, Austria) to examine standardized intervention vs. control group differences in (a) post- vs. pre-intervention changes and (b) post-follow-up vs. pre-intervention changes of depression scores [20]. Additional subgroup analyses were conducted to examine these differences based on the instruments used to measure depression (e.g. Beck Depression Inventory-II (BDI-II) and Center for Epidemiologic Studies Depression Scale (CES-D)). The consistency of the findings of the meta-analyses was confirmed by leave-one-out sensitivity analyses [21]. Given that under-reporting and publication bias could result in biased (i.e., smaller) estimates, publication bias was examined using funnel plots and with the aim of imputing effect sizes of missing (i.e., unpublished/unreported) studies via the trim-and-fill method [22,23]. Heterogeneity of effect sizes was quantified by calculating the Higgins’ I2 statistic [24,25]. To explain the heterogeneity of the studies, exploratory univariate random-effects meta-regression analyses were performed to examine the moderator effects of each of the covariates described above [26].

Results

Initial Search Results

The title search on PubMed, Clinical Key, and Web of Science yielded 235 articles after excluding those that did not mention positive psychology or depression. The abstract screen yielded 39 articles after excluding articles that did not use PPI. Full texts were assessed and reviewed individually, yielding 10 articles (see Figure 4 for a breakdown). A secondary search was conducted through relevant literature reviews and meta-analyses, and two articles were included in this study as hand-picked articles. During the primary meta-analysis step, one article was eliminated to minimize overlap due to similarity in protocol and results, which did not significantly change the pooled prevalence estimate. There were 11 total articles assessed in the final meta-analysis [12,27-36].

Critical Appraisal

The quality assessment included whether the article was peer-reviewed, was a randomized controlled trial, double-blinded, or included a follow-up (Table 1).

**Table 1:** Quality assessment.

Article	Blinded	Peer-reviewed	Randomized	Follow-up	Control condition
1. Bolier et al. (2013) [27]		✓	✓	✓	✓
2. Chaves et al. (2017) [28]	✓	✓			✓
3. Ducasse et al. (2018) [12]	✓	✓	✓		✓
4. Furchtlehner et al. (2019) [29]		✓	✓	✓	✓
5. Guo et al. (2017) [30]		✓	✓	✓	✓
6. Hanson (2019) [31]		✓	✓	✓	✓
7. Proyer et al. (2014) [32]		✓	✓	✓	✓
8. Ramirez et al. (2014) [33]		✓	✓	✓	✓
9. Asgharipoor et al. (2012) [34]		✓	✓		✓
10. Silton et al. (2020) [35]		✓		✓	
11. Celano et al. (2017) [36]	✓	✓	✓	✓	✓

Results From the Literature

Table 2 summarizes the 11 articles analyzed in this study. Collectively, 736 participants were in the PPI group and 615 participants were in various control groups. There were three main categories of PPIs among the 11 studies, and they included internet-based interventions (2), group positive psychotherapy (5), and individual reflection or self-help (3). A majority of the group therapy interventions were weekly and also included homework and exercises between sessions.

**Table 2:** Summary of studies included. CES-D: Center for Epidemiologic Studies Depression Scale; DSM-IV: Diagnostic and Statistical Manual of Mental Disorders, Fourth Edition; SCID: Structured Clinical Interview for DSM Disorders; C-SSRS: Columbia Suicide Severity Rating Scale; MDD: major depressive disorder; MINI: Mini International Neuropsychiatric Interview; PP: positive psychology; CBT: cognitive-behavioral therapy; PPI: positive psychology intervention; CF: cognition-focused.

Study	Country	Delivery	Sessions, duration	N	Clinical status	Age range	Control group	Findings
1. Bolier et al. (2013) [27]	Netherlands	Psyfit, internet-based intervention, personal training program	6 modules, 2 months	143	Mild to moderate depression (CES-D)	Mean: 43.5	Waiting list	Significant improvement (p = 0.049) in overall well-being with intervention according to WHO-5 measurement for two-month follow-up
2. Chaves et al. (2017) [28]	Spain	In-person group psychotherapy with a licensed therapist	Weekly 2-hour sessions, 10 weeks	47	Women with major depression or dysthymia (DSM-IV, SCID)	Mean: 52.57	CBT	CBT and PPI are equally effective in treating depressive symptoms (p = 0.84)
3. Ducasse et al. (2019) [12]	France	Gratitude diary with individual reflection	7 days	101	Patients hospitalized with MDD and suicide ideations (C-SSRS)	18-65	Food diary	PPI significantly improved levels of depression in comparison to the control group (p = 0.008). Gratitude was associated with improved optimism
4. Furchtlehner et al. (2019) [29]	Austria	Group psychotherapy with a licensed therapist using the PERMA model	14 weeks	46	Depressive disorder (DMS-IV)	18-60	CBT	PP patients demonstrated fewer depressive symptoms over six months compared to CBT patients (p < 0.001)
5. Guo et al. (2017) [30]	Australia	Group psychotherapy with eight exercises	1.5 hours discussion and 1-week practice per exercise, 8 weeks	42	Nursing students with mild-moderate depression (BDI-II)	Mean: 20.39	School routine help (once a semester psychological counseling)	The intervention significantly alleviated depression (p = 0.0001) with significantly lower scores than the control group. PPT cultivated positive thoughts and behavior, which is associated with less depressive symptoms
6. Hanson (2019) [31]	England	PP self-help book titled positive psychology for overcoming depression	8 weeks	16	Depressive symptoms not receiving treatment	19-69	CBT self-help book	There is no statistical difference (p = 0.5) in PP and CBT self-help books, but bibliotherapy is a cost-effective method to alleviate depressive symptoms
7. Proyer et al. (2014) [32]	Switzerland	Four online and self-administered exercises: gratitude visit, three good things, using signature strengths, three funny things	1 week	163	Depressive symptoms	50-70	Early memories	Three interventions (gratitude visit, three good things, and signature strengths) statistically increased happiness, and two interventions (three funny things and signature strengths) reduced depressive symptoms
8. Ramirez et al. (2014) [33]	Spain	Group psychotherapy with homework via MAPEG program	1.5 hour sessions per week, 9 weeks	26	Depressive symptoms	60-93	Placebo (early memories)	The experimental group demonstrated a significant decrease in state anxiety and depression as well as an increase in specific memories, life satisfaction, and subjective happiness, compared with the placebo group (p < 0.04)
9. Asgharipoor et al. (2012) [34]	Iran	Group psychotherapy with citalopram medication (20-40 mg)	12 weeks	9	MDD	Mean: 36	Group CBT with citalopram medication (20-40 mg)	PPI is more effective in increasing happiness in MMD patients than CBT
10. Silton et al. (2020) [35]	USA	Savoring intervention with individual reflection	1 week	111	Depressive symptoms	Mean: 70.7	None	PPI was successful at reducing depressive symptoms in those participants with high fidelity
11. Celano et al. (2017) [36]	USA	HOPE, treatment manual with PP exercises and weekly one-on-one telephone sessions	6 weeks	32	Patients with MDD recently hospitalized for suicidal ideation or attempt (MINI)	Mean: 43.2	Cognition-focused intervention	PPI was successful at increasing optimism and gratitude and decreasing suicide ideation and depression. However, it was not superior to the CF control

Post- vs. Pre-intervention Difference

**Figure 5:**
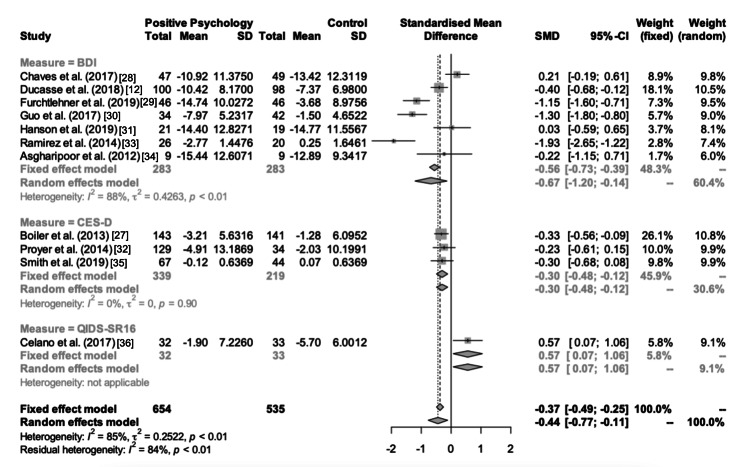
Forest plot depicting differences in depression scores for post vs. pre-intervention for each study.

In the random-effects meta-analysis that compared PPIs vs. control groups on post- vs. pre-intervention differences in depression scores, PPIs were observed to significantly decrease standardized depression scores compared to the controls (pooled Cohen’s d = −0.44 (−0.77, −0.11)) (Figure 5). Excluding any single study from the meta-analysis (i.e., leave-one-out sensitivity analyses) did not significantly change the pooled prevalence estimate. Subgroup analyses performed to examine the pooled post- vs. pre-intervention differences within the studies using BDI-II and CES-D also revealed significant pooled post- vs. pre-intervention differences (pooled Cohen’s d = −0.67 (−1.20, −0.14) and pooled Cohen’s d = −0.30 (−0.48, −0.12), respectively). Only one study used Quick Inventory of Depressive Symptomatology-Self-Report (QIDS-SR16) and it showed a significant increase in depressive symptoms with PPI compared to the control intervention (Cohen’s d = 0.57 (0.07, 1.06)). Furthermore, the funnel plot of effect sizes was symmetrical, indicating publication bias seems to be less likely.

Significant heterogeneity was observed among the mean differences pooled in the random-effects model (τ2 = 0.252; I2 = 85.3%; p < 0.001). Exploratory univariate random-effects meta-regression analyses conducted with the aim of explaining the heterogeneity using the moderator effects of the considered covariates suggested the increased proportion of individuals engaged in higher education had a trend in decreasing the intervention effect (p = 0.077) and the measure QIDS-SR16 was also associated with a significant decrease in the PPI effect (p = 0.036). Yet, these covariates failed to significantly explain the heterogeneity of effect sizes. All other covariates did not show significant moderator effects on univariate meta-regression analyses (p > 0.050). The combination of mean age, year of publication, the measure of depression, and the proportion involved in higher education collectively explained the 86.43% of residual heterogeneity (τ2 = 0.0019; I2 = 33.1%; p < 0.002). In this model, mean age was a significant positive moderator (p = 0.008), suggesting that increasing age was associated with a decreased intervention effect. The year of publication was a significant negative moderator (p = 0.012), suggesting that the effectiveness of PPIs may have improved with time. The other two covariates did not have a significant moderator effect.

Follow-Up- vs. Pre-intervention Difference

**Figure 6:**
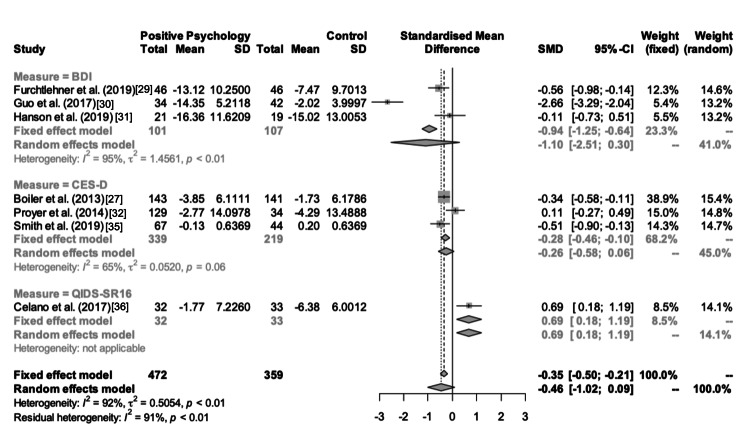
Forest plot depicting differences in depression scores for follow-up vs. pre-intervention for each study.

In the random-effects meta-analysis that compared PPIs vs. control groups on post-follow-up- vs. pre-intervention differences in depression scores, PPIs were observed to decrease standardized depression scores compared to the controls (pooled Cohen’s d = −0.46 (−1.02, 0.09)) (Figure 6); however, this reduction was not statistically significant. Leave-one-out sensitivity analyses revealed that excluding the Celano et al.'s [36] study from the meta-analysis reveals a significant treatment effect for PPI (Cohen’s d = −0.64 (−1.21, −0.09), p = 0.0238). Subgroup analyses performed to examine the pooled post- vs. pre-intervention differences within the studies using BDI-II and CES-D, however, did not reveal significant differences, possibly due to the limited number of studies in each group (pooled Cohen’s d = −1.10 (−2.51, 0.30) and pooled Cohen’s d = −0.26 (−0.58, 0.06), respectively). On the other hand, Celano et al. [36] showed a detrimental effect on depression symptoms with PPI compared to the control intervention (Cohen’s d = 0.69 (0.18, 1.19)). Funnel plot of effect sizes was evident of potential publication bias, yet the trim-and-fill method failed to impute missing effect sizes.

As in the previous meta-analysis, significant heterogeneity was a concern (τ2 = 0.505; I2 = 92.2%; p < 0.001). Exploratory univariate random-effects meta-regression analyses revealed a negative moderator effect of the proportion of individuals engaged in higher education (p = 0.011), suggesting that the PPIs seem to be more effective for individuals who have received higher education. Yet, this covariate failed to significantly explain the heterogeneity. Mean age had a positive trend on the pooled standardized mean difference, suggesting that the effectiveness of the PPIs on depression seems to decrease with age. All other covariates did not show significant moderator effects on univariate meta-regression analyses (p > 0.050). The combination of the proportion involved in higher education and the depression scale fully explained the residual heterogeneity (τ2 = 0.000; I2 = 0.0%, p < 0.001). In this model, the proportion that received higher education remained a significant negative moderator (p < 0.001) and the CES-D scale (compared to the reference, BDI-II) remained a significant positive moderator (p < 0.001).

Discussion

Main Findings From the Literature

This study is the first meta-analysis conducted that analyzed the efficacy of PPIs on the treatment of depressive symptoms with high-quality studies. The results from pooling the effect sizes together in a random-effects model show that compared to a variety of different control groups, PPIs provided significantly decreased depression scores in both post-treatment and post-follow-up measures.

The study found significant heterogeneity for both the follow-up and pre-intervention groups, as well as in the pre- and post-intervention differences, which indicate that positive psychology worked very well in some studies and moderately well in others. The diversity in the methods and duration of PPIs used across the studies, ranging from weekly group therapy to daily self-journaling, accounts for the heterogeneity found in this meta-analysis. Apart from one study [36], all studies found PPIs to be more effective and beneficial in participants compared to the control. The participants in this study were acutely depressed and reported suicidal ideation or a recent suicide attempt. Due to the severity of their symptoms, this group of patients may have found it difficult to actively engage in positive psychology exercises. This suggests that positive psychology may not be suitable for higher-risk or severely depressed patients who are unable to actively engage in positive thoughts.

All but one of the 11 studies had a control group [35]; the control groups included a CBT intervention, placebos, and a waiting list. CBT has extensive empirical evidence and was used in control groups for half of the studies. An important finding was from Lopez-Gomez et al.'s study [37], where it was shown that patients who had positive psychology had much better outcomes than CBT treatment [37]. This implies that PPI is beneficial for most patients, and for patients who are not suited for other forms of psychotherapy, PPI may provide the optimal treatment. Further studies can inform which populations would optimally benefit from PPI.

Analysis of the Covariates

Multiple covariates, such as the proportion of females and education level, were analyzed to see whether or not they could explain the heterogeneity between the studies. The proportion of females in a study group and the proportion of individuals engaged in higher education were two covariates that increased the intervention effect, meaning that PPIs tend to work better for females and individuals with higher levels of education. While this is an association, and not causal, this is an important implication to keep in mind when assessing patients for mental health treatment options. Mean age was also found to be a significant positive moderator, and PPIs tended to work better for older individuals. For unclear reasons, older publications, regardless of intervention utilized, had greater effect sizes.

Relevance to Clinical Practice

This meta-analysis has important implications for clinical practice in treating depression as it clearly highlights the efficacy of PPI. Currently, the most utilized approach for treating depression is psychopharmacology with results being far from satisfactory. Psychotherapy, while considered first-line treatment, has time and monetary constraints. Resistant cases of depression are usually referred for repetitive transcranial magnetic stimulation (rTMS) and electroconvulsive therapy (ECT). Those treatments also have constraints in addition to being not available for many patients [38-41]. Thus, there is a need for alternative treatment options that are efficacious and accessible.

PPIs can be a valuable modality as a sole treatment as well as an adjunctive one. For example, in a meta-analysis conducted in 2018, 21 antidepressant drugs were tested on people with major depressive disorder (Cohen’s d = 0.30), and its pooled effect size was lower compared to this meta-analysis using positive psychology (Cohen’s d = −0.54) [42]. CBT and PPI are often compared, and it has been shown that some people respond better to PPI whereas others show greater improvements with CBT. Since PPIs work well even when self-administered, there is less of a need for continuous physician support, which allows individuals to have long-term agency in maintaining their treatment.

The results from this study also have implications for primary care settings. Often, primary care physicians are the first to see psychological problems or co-morbidities in patients, which explains the description of primary care being the de facto mental health services system [43]. Not only is there an increasing demand for primary care physicians to handle mental health crises, but there is also a lag time between a primary care visit and a psychiatrist referral; studies show that less than one-third of mental health referrals are actually completed [44]. Because many PPIs are easily administered, and can even be self-administered, patients with less severe symptoms could be treated within a primary care setting without needing to be seen by a specialist. This makes mental health treatment more streamlined and it would better integrate physical and mental health maintenance with very little extra cost or energy investment [45].

Many of the interventions in this study were group positive psychotherapy. A potential reason for the popularity of group therapy is that it is more cost-effective than individual therapy, and individuals can learn from each other and the group dynamic. Online-administered modules and self-help interventions are even less costly, and they allow for more schedule flexibility for busy adults [31]. This way, mental health treatments can reach a broader audience. Additionally, with the rise in the popularity of telemedicine, there may be an increase in efforts to improve and develop internet-based interventions [46]. PPI is less commonly used in practice with healthcare providers, including psychiatrists, who generally do not get sufficient education, if any at all, on PPI during medical school or residency training [47]. Despite this challenge, there are existing methods for implementing PPI in clinic settings. For example, clinicians can use the Values in Action Inventory of Strengths (VIA-IS), which assesses a patient's top five strengths so that clinicians can help patients develop weekly goals to utilize these strengths [48]. This is just one of the many simple assessments described in a study by Slade; this study challenges clinicians to shift away from the emphasis on patient deficits and problems that most psychiatric histories focus on [48]. If patients are encouraged to pay more attention to worthy traits, they can have a more balanced and holistic view of themselves, which facilitates healing.

Criticisms of Positive Psychology

Critics of the positive psychology center their argument on the importance of knowing the limitations of positive psychology, the distinctiveness of the movement, and the ambiguous meaning of what is good [49]. It is important for patients undergoing therapy to recognize the limitations of positive psychology. Many critics of positive psychology claim that it serves to erase the negative realities of a situation, but that is not the approach taken by PPIs [50]. PPIs instead focus on building strength and resilience to help patients face their realities. It emphasizes a balanced mindset since it is difficult to stay optimistic while struggling with physical or mental illnesses. For example, patients with chronic diseases such as cancer or dementia may benefit from positive thinking but must also cope with their realities [18].

Many critics of positive psychology believe that if positive psychology exists, then the rest of the field should be considered negative psychiatry. In fact, psychology is mostly neutral but usually is centered around negative dilemmas [49,51]. One final critique is that due to the ambiguous nomenclature of what is good and positive, it is difficult to standardize study findings and draw conclusions about positive psychology’s efficacy. Proponents of positive psychology believe that cultural norms or value systems can determine what is good.

Limitations

The studies included in this meta-analysis had limitations regarding sample size, study duration, and sample population heterogeneity. For example, three out of 11 of the studies had a sample size of less than 30 individuals [31,33,34]. Two of the 11 studies reported in this meta-analysis consisted of only women participants [28,32]. Furthermore, three of the 11 studies analyzed were only weeklong [12,32,35]. Outcomes of this meta-analysis could be potentially biased due to experimenter bias of individual studies. However, there is a statistically low publication bias in the field of positive psychology and therefore in this meta-analysis. Heterogeneity is expected in any meta-analysis because of variability in studies, and this meta-analysis was not resistant due to variability of methodology and study samples. Future studies should be held for a long duration, with larger sample size, and that study the general population more comprehensively. Finally, although this study focused on the effects of PPIs, the participants of some of these studies were also taking pharmacological intervention, which could play the part of a confounding variable in this study. However, we included studies where the pharmacologic agent was utilized in both the intervention and control groups.

Future Directions

Future directions to build on this study include finding the efficacy of PPI on different populations. For example, testing the efficacy of PPI in treating depression in adolescents could be an interesting find as this analysis only included studies with adult participants over the age of 18 years. More specific studies can also be done that address the efficacy of positive psychology on depressed patients from varied races, socioeconomic classes, education levels, and health statuses. While this study focused on treating depression and depressive symptoms, a future direction could be analyzing more severe mental disorders that could benefit from PPIs, such as schizophrenia or bipolar disorder. Expanding the possibilities would mean increasing the patient population that could benefit from these interventions.

## Conclusions

This paper is the first meta-analysis on the efficacy of positive psychology on depression in the last five years. The results from this analysis show a promising future for the field of positive psychology and its plethora of interventions. This meta-analysis demonstrates positive psychology is efficacious in treating most patients with depression or depressive symptoms. With a focus on increasing individual well-being and agency, while not neglecting the negative aspects of mental illness, PPIs equip individuals with a balanced set of tools for coping with adversities in life. While there is still much to learn about the precise mechanisms and factors that it functions by, one thing is clear: people are benefiting from positive psychology and we should continue to advocate for its use and growth.
